# International Collaboration for the Epidemiology of eGFR in Low and Middle Income Populations - Rationale and core protocol for the Disadvantaged Populations eGFR Epidemiology Study (DEGREE)

**DOI:** 10.1186/s12882-016-0417-1

**Published:** 2017-01-03

**Authors:** Ben Caplin, Kristina Jakobsson, Jason Glaser, Dorothea Nitsch, Vivekanand Jha, Ajay Singh, Ricardo Correa-Rotter, Neil Pearce

**Affiliations:** 1 Centre for Nephrology, UCL Medical School Royal Free, London, NW3 2PF UK; 2 Division of Occupational and Environmental Medicine, Sahlgrenska Academy, Gothenburg University, Gothenburg, Sweden; 3 Division of Occupational and Environmental Medicine, Lund University, Lund, Sweden; 4 La Isla Foundation, Ada, Michigan USA; 5 Department of Non-Communicable Disease Epidemiology, London School of Hygiene and Tropical Medicine, London, UK; 6 George Institute for Global Health India, New Delhi, India; 7 University of Oxford, Oxford, UK; 8 Department of Global and Continuing Education, Harvard Medical School, Boston, USA; 9 Department of Nephrology and Mineral Metabolism at the Instituto Nacional de Ciencias Medicas y Nutricion Salvador Zubirán, Mexico City, Mexico; 10 Department of Non-Communicable Disease Epidemiology and Centre for Global NCDs, London School of Hygiene and Tropical Medicine, London, UK

**Keywords:** Chronic kidney disease of undetermined cause, Low- and middle-income countries, Mesoamerican nephropathy, Estimated glomerular filtration rate, Chronic kidney disease prevalence

## Abstract

**Background:**

There is an increasing recognition of epidemics of primarily tubular-interstitial chronic kidney disease (CKD) clustering in agricultural communities in low- and middle-income countries (LMICs). Although it is currently unclear whether there is a unified underlying aetiology, these conditions have been collectively termed CKD of undetermined cause (CKDu). CKDu is estimated to have led to the premature deaths of tens to hundreds of thousands of young men and women over the last 2 decades. Thus, there is an urgent need to understand the aetiology and pathophysiology of these condition (s). International comparisons have provided the first steps in understanding many chronic diseases, but such comparisons rely on the availability of standardised tools to estimate disease prevalence. This is a particular problem with CKD, since the disease is asymptomatic until the late stages, and the biases inherent in the methods used to estimate the glomerular filtration rate (GFR) in population studies are highly variable across populations.

**Method:**

We therefore propose a simple standardised protocol to estimate the distribution of GFR in LMIC populations – The Disadvantaged Populations eGFR Epidemiology (DEGREE) Study. This involves the quantification of renal function in a representative adult population-based sample and a requirement for standardisation of serum creatinine measurements, along with storage of samples for future measurements of cystatin C and ascertainment of estimates of body composition, in order to obtain valid comparisons of estimated GFR (eGFR) within and between populations.

**Discussion:**

The methodology we present is potentially applicable anywhere, but our particular focus is on disadvantaged populations in LMICs, since these appear to be most susceptible to CKDu. Although the protocol could also be used in specific groups (e.g. occupational groups, thought to be at excess risk of CKDu) the primary aim of the DEGREE project is characterise the population distribution of eGFR in multiple regions so that international comparisons can be performed. It is only with a standardised approach that it will be possible to estimate the scale of, and variation in, impaired kidney function between affected areas. These data should then provide insights into important social, demographic and environmental risk factors for this increasingly recognised disease.

**Electronic supplementary material:**

The online version of this article (doi:10.1186/s12882-016-0417-1) contains supplementary material, which is available to authorized users.

## Background

Chronic impairment of kidney function is most commonly associated with diabetic nephropathy, vascular disease, glomerulonephritis, congenital abnormalities or obstruction of the urinary tract. Although such impairment is usually asymptomatic in the early stages, the prevalence of end-stage renal disease requiring renal replacement therapy is increasing worldwide [1]. Much of this increase can be attributed to an increasing prevalence of known risk factors. However, there is now also increasing recognition of forms of progressive kidney injury which are not associated with diabetes, vascular disease, or glomerulonephritis, and which are affecting the working-age populations of low- and middle- income countries (LMICs).

This chronic impairment of kidney function not associated with known risk factors or a specific histological diagnosis has been termed CKD of undetermined cause (CKDu). Other terms used include CKD of non-traditional cause (CKDnt) and Mesoamerican nephropathy (MeN) when describing the condition in parts of Latin America. For the purposes of this paper we will use the term CKDu. Clusters of CKDu occur in primarily (but perhaps not exclusively) in communities characterised by a hot climate and reliance on heavy agricultural work. Although various causal and contributing factors have been proposed there is currently no definitive evidence for the role of a specific aetiological pathway. CKDu carries a poor prognosis, as renal replacement therapy is often inaccessible to the majority of the population in many of the affected areas. Over the last two decades, clusters of CKDu have been reported in Central America [2] and Sri Lanka [3]. Other reports have suggested that similar patterns may be occurring in regions of India [4], Saudi Arabia [5], Egypt [6], and Senegal [7]. However, significant difficulties arise in comparing studies conducted in different regions due to differing study designs, sampling approaches and case-definitions. Furthermore, the impact of CKDu on those affected, and the resulting strain on health systems, makes the estimation of CKDu prevalence a global health priority.

In the past, international comparisons have played a key role in identifying possible causes of chronic disease [8]. For example, many of the discoveries on the causes of cancer (including dietary factors and colon cancer, hepatitis B and liver cancer, aflatoxins and liver cancer, human papilloma virus and cervical cancer) have their origins, directly or indirectly, in the systematic international comparisons of cancer incidence conducted in the 1950’s and 1960’s. These suggested hypotheses concerning the possible causes of the international patterns, which were investigated in more depth in further studies. In some instances these hypotheses were consistent with biological knowledge at the time, but in other instances they were new and striking, and might not have been proposed, or investigated further, if the population level analyses had not been done.

A more recent example of the value of international comparisons and collaborations is the International Study of Asthma and Allergies in Childhood (ISAAC) a standardised protocol to estimate the prevalence of asthma internationally [9, 10]. The most recent phase (Phase Three) has involved 1,187,496 children in 237 centres in 98 countries [11–13]. This has led to greater understanding of the possible causes of asthma globally, as well as the creation of a large international network of researchers. We propose a similar model involving a simple and practical protocol to estimate distributions of renal function in disadvantaged communities globally: the Disadvantaged Populations estimated Glomerular Filtration Rate (eGFR) Epidemiology Study (DEGREE). This will provide key information to inform hypotheses and to guide further research into the causes of CKDu.

### Challenges

A number of challenges arise when attempting to obtain standardized estimates of CKDu prevalence globally:

1. CKDu is usually clinically silent until it has progressed to advanced stages, so the diagnosis of mild renal impairment must be made by testing asymptomatic individuals.
2. Awareness of CKDu is highly variable so attempts to determine disease burden using routine healthcare data or mortality records may be misleading.
3. Variation in access to renal replacement therapy both within and between regions means that dialysis or transplant registry data are also unlikely to reflect disease prevalence.
4. Unlike CKD due to causes such as diabetes or glomerulonephritis, CKDu is associated with a low prevalence of hypertension and significant proteinuria, at least in the early stages. Therefore commonly instituted mass non-communicable disease screening tools such as blood pressure measurement or dipstick urinalysis have poor sensitivity for detecting cases of CKDu in a population.
5. Renal biopsies are unlikely to be performed systematically in affected populations; where these have been undertaken they have generally not demonstrated specific findings, making tissue diagnosis of CKDu difficult. Similarly, ultrasound and other types of renal imaging are usually non-informative.
6. The distribution of the GFR and the extent to which this measure predicts end-stage renal disease is not well characterised in the affected populations and may vary between regions.
7. Estimates of GFR from serum markers such as creatinine, e.g. using the eGFR calculated by either the Chronic Kidney Disease Epidemiology Collaboration (CKD-EPI) or Modified Diet in Renal Disease (MDRD) equations, become more imprecise as the level of GFR approaches normal [14].
8. The degree of bias in these estimates of GFR is also a function of ethnicity, and body weight/muscle mass [14], and, the precision of ethnicity-adjusted equations is still highly variable across geographic regions [15] rendering international comparisons particularly problematic.
9. GFR estimation based on serum markers is critically dependent on adequate standardization of assays [16].
10. Finally, the definition of CKDu is currently one of exclusion. The causes to be excluded may change over time as our knowledge increases. Thus, the various phenotypes, and their definitions, may change over time.

### Rationale

For the above reasons, the protocols presented here are not intended to yield definitive information on CKDu prevalence. Rather, they are intended to yield standardized estimates of the distribution of eGFR, and hence the prevalence of reduced renal function, a precursor of CKD in general, and CKDu in particular. Since definitions of CKDu are currently controversial and changing (in particular, this is a definition by exclusion, and may be refined in future), the aim is not to definitely estimate the prevalence of CKDu itself, but rather to obtain the key information that can be used in a variety of definitions of CKDu in future. Nevertheless, we of course also propose collecting information on other causes of CKD (e.g. diabetes), and use these (by exclusion) to identify possible cases of CKDu, and more generally of impaired renal function which is not due to known causes.

It is clear that differences in creatinine assay calibration have contributed to a large proportion of the total variability in reported eGFR values in epidemiological studies to date [17]. Failure to use standardised serum creatinine values for international comparisons would therefore lead to quantification of variability in laboratory practice rather than comparisons of eGFR distributions. Therefore any eGFR data submitted to the DEGREE project must use serum creatinine values that are measured by assays calibrated using quality controls traceable to an isotope dilution mass spectrometry (IDMS) standard. Calibration results and quality control information will also need to be submitted with study data.

It should be recognised that there is no single validated easily deliverable solution to the issue of differential bias in GFR estimating equations between populations/ethnic groups. In the DEGREE protocol this problem will be addressed by two main approaches to allow us to perform meaningful international comparisons, these are: (i) storage of samples for centralised cystatin C measurement and (ii) collection of estimates body composition. In addition we hope to support the performance of measured GFRs in subpopulations in selected centres. Although both sample storage for central cystatin C assays and bioimpedence measurements are currently included in the core protocol it may be that as the DEGREE project grows, alongside the validation of eGFR equations in different populations, these aspects of the protocol can be further simplified.

Cystatin C is an endogenous marker of kidney function less dependent on muscle mass. Both costs and lack of standardisation across different laboratories [18] mean that locally performed cystatin C measurements are not currently suited to international comparisons. Furthermore although methods based on this test have generally been shown to improve the precision of eGFR measurements [19], there are other non-renal sources of variation in cystatin C [20], and this approach has not yet been validated in many of the populations of interest. However with collaborative efforts aimed at both assay standardisation and validation, cystatin C based eGFR estimation is likely to go on to become the marker on which eGFR is calculated locally in the future. Meanwhile the DEGREE committee plans to provide the opportunity to measure cystatin C centrally for a number of centres and therefore storage of samples for this purpose is included in part of the core protocol.

The second approach uses diet and body composition data, which will be collected in parallel as part of the protocol using single frequency 50Hz bioimpedence devices along with self-reported meat consumption. Adjustment of measures of creatinine based renal clearance for estimates of creatinine production have been shown to provide a more precise estimate of excretory function across a range of physical builds and renal function [21, 22]. Simple bioimpedence devices produce highly reproducible measurements, are now routinely in use by nutritional researchers globally and are available cheaply. Although body composition estimates from bioimpedence data may also be subject to ethnicity-dependent bias, the magnitude of this measurement error can be minimised using a simplified approach and is substantially smaller than that observed with GFR estimating equations [23, 24].

The DEGREE committee are keen that investigators are not dissuaded from performing studies by the inclusion of the above aspects in the protocol. Therefore any research group keen to undertake a DEGREE study, but feel they are unable to store samples for later cystatin C estimation and/or collect body composition data are advised to contact the DEGREE executive.

Finally, although it is recognised that these procedures will be beyond the resources of many centres, we also hope some research teams will be interested in performing measured GFRs using simplified iohexol clearance techniques in a subsample of the population in some centres [25]. These procedures will provide insight into the validity of GFR estimates across the range of kidney function using endogenous markers in the relevant populations. A number of research teams are pursuing this type of study independently however the executive would be keen to support investigators to perform these protocols in the populations that are the focus of the DEGREE project. Interested centres can contact the DEGREE executive.

### Aims

Taking the above challenges and rationale into account the aims of the DEGREE collaboration are to:

1. Conduct representative surveys of the distribution of eGFR in populations in different regions and to make regional and international comparisons.
2. Estimate the proportion of those with reduced eGFR but without diabetes, hypertension or proteinuria in each population and compare this between regions and internationally.
3. Generate baseline data so that future shifts in the eGFR distribution in affected areas can be determined.
4. Provide the basis for future standardised studies, including but not limited to: detailed phenotypic examinations, environmental investigations, quantification of occupational exposures, cohort studies and interventional trials
5. Establish a framework for international collaboration and foster an environment for further work into the prevalence and causes of CKDu.

## Design

### Organisation of the DEGREE project

The DEGREE project is an international collaboration involving standardized protocols for estimating population patterns of eGFR. Individual centres can register with the DEGREE study by submitting a registration document (Additional file 1). A number of study sites have either commenced, or are in the planning stages of, i.e. awaiting the outcome of funding decisions, studies with protocols using the DEGREE methodology (with minor modifications in some cases). Centres will be required to follow the standardised core protocol in order to be part of the DEGREE collaboration, and to be included in international comparisons. However, investigators will be encouraged to enhance this minimum protocol with additional investigations as appropriate to the local situation. Possible examples include: additional questions focused on work exposures, renal ultrasound imaging, formal albumin:creatinine ratio measurements. Example proformas for some of these additional modules are available in the Additional file 2 or from the authors.

### Core protocol - ethical and regulatory issues

Participants will need to provide informed consent before taking part in the study, and Investigators will be responsible for producing participant information sheets and gaining approval from the local institutional review board. It is envisaged that eGFR results will be communicated back to participants once available. The need for repeat testing in those subjects with raised serum creatinine levels, the impact of abnormal findings on those taking part (including newly diagnosed low eGFR, glycosuria or hypertension), as well as mechanisms for onward referral to local healthcare systems will constitute an important aspect of the ethical framework in which this protocol is undertaken. These issues should be formalised into the local version of the protocol before commencing a DEGREE study. Furthermore, the need for close collaboration with the local and national health system during the planning phase of a DEGREE study includes not only the system for individuals in need of follow up, but also agreements on how results from the DEGREE study at the population level should be communicated to different stakeholders for public health purposes.

### Core protocol - questionnaires and study instruments

The aims of the core questionnaire and clinical measurements are to obtain a minimum core dataset to allow the estimation of the distribution of eGFR within and between populations, and to estimate the prevalence of reduced GFR that is not due to diabetes, glomerulonephritis or associated with hypertension. Investigators should add additional questions on exposures that are relevant to the local context or concerns of the population. The questionnaires and protocols (see Additional file 3) have been developed by modification of the STEPS instruments [26]. Translation and validation of questionnaires into other languages so that all groups within a region can participate will be the responsibility of local coordinators. Questionnaires should be conducted in a language spoken by all participants or translators recruited where participants may not all speak the same language.

### Core protocol - population and sampling strategy

For robust international comparisons a representative population is critical. The protocol should be undertaken in randomly–selected sample (or alternatively the entire population) of adults aged over 18 years in a specified geographical area (i.e. study participants must not be selected based on them presenting to healthcare facilities or advertised screening sessions). A region or district should be selected and clearly defined using GIS coordinates or map. Sampling units can be individuals or households; in the former case, a new census should be conducted unless an up-to-date and complete census is already available for the entire defined area. This protocol may of course also be applied in specific populations, for example occupational groups, but studies based on non-representative samples will not be used for international comparisons in the first phase of the DEGREE project.

The ideal sample size for a population-based study (see below) is 1000 participants per study centre, and this is required for inclusion in international comparisons, but it is recognised that studies smaller than this and in specific populations, particularly in occupational groups, may generate valuable information.

### Core protocol - study visits

The study visit will involve the administration of the core questionnaire, basic clinical measurements, dipstick urinalysis and a blood draw for serum creatinine measurement (proformas for these are included as Additional file 3). Where possible participants should be asked to attend first thing in the morning and fasted overnight, i.e. before the first meal of the day and before starting work.

### Core protocol - testing

Dipstick urinalysis should be performed using commercial testing sticks according to manufacturers instructions. Sticks should report urine blood, protein (at macroalbminuric level), leucocytes, glucose, pH and specific gravity as a minimum and ideally read using an optical reader. Serum creatinine should be measured locally using a method calibrated to an isotope dilution mass spectrometry standard with assay and calibration details reported to the DEGREE data centre. Height should be measured using a stadiometer and weight using digital scales. Body composition should be quantified using calibrated single frequency (50 kHz) bioimpedence analysis instrument (supine instrument where possible) as a direct impedance output, with machine-calculated values also reported (examples and suppliers of suitable instruments can be obtained from the DEGREE executive). Additional bio-samples (e.g. whole blood, serum and urine) should be collected and stored at -20C (or lower where possible) to allow for cross-centre validation of serum creatinine values and the testing of additional markers of renal function such as cystatin C as discussed above. The number and type of these samples will depend on local circumstances but we suggest a minimum of one additional serum sample (total 5 mL stored as three 1.5 mL aliquots) for each participant. These samples should be handled in accordance with local standard operating procedures with appropriate attention to labelling, storage, equipment maintenance and documentation [27].

### Core protocol - data management and reporting

All DEGREE protocols are freely available to interested investigators. Each individual Centre will ‘own’ their own data, but will be asked to submit an anonymised copy of individual level data, together with contextual information on the investigated population to the DEGREE Data Centre for inclusion in international comparisons.

Paper based studies should use double data-entry to minimise human manual transcription error. Samples and questionnaires will ideally be identified using a barcode system. Centres can submit their cleaned data using sample database available from the DEGREE team based in London.

A number of ongoing projects are currently attempting to assess the performance of eGFR estimating equations in different regions and developing ethnicity specific modifications. Until these are available, data should be provided as serum creatinine estimates to enable standardised calculation at the analysis centre with a variety of formulae.

### International comparisons

International comparisons will be conducted and coordinated by the DEGREE Steering Committee. Descriptive statistics of eGFR using both CKD-EPI and MDRD formulae (with and without adjustment for body composition and self-reported meat consumption) will be presented by country, region, age and sex. The distributions of eGFR will be estimated in the full sample, and then restricted to those without evidence of hypertension, diabetes, or urinary evidence of glomerulopathy. As secondary analyses we will investigate the prevalence of people with eGFR < 90 ml/min restricted to those without hypertension, diabetes, or urinary evidence of glomerulopathy, adjusted for age, sex and body composition.

Reporting to the DEGREE data centre should be in accordance with previous guidelines [28]. This includes description of the sampling frame, the characteristics of the population, the sampling method, response rates and the methodology used for laboratory and clinical measurements. Example reporting forms are presented in the Additional file 3.

### Study size and power

The required study size has been estimated based on two considerations: (i) obtaining reasonably accurate estimates of eGFR in each centre; (ii) having sufficient statistical power for comparisons between centres or between population subgroups. For both these considerations, the power calculations have been done two ways: (i) using eGFR as a continuous variable (mean, sd) in order to estimate the population prevalences of impaired kidney function and (ii) ‘cutoffs’ for eGFR. The overall aim is to have sufficient statistical power to estimate prevalence, and detect population differences of epidemiological and health service significance.

Overall, a recommended sample size of 1,000 participants has been chosen.

Estimates within a single population: Using a continuous measure if the ‘true’ mean eGFR is 110 mL/min (although it is recognised this value is likely to vary substantially between different regions), with a standard deviation (SD) of 30 mL/min it is more than 95% likely that the estimated mean will be between 108 mL/min and 112 mL/min. Assuming a ‘true’ prevalence of eGFR <90 mL/min of 5%, then with a sample size of 1,000, it is 95% likely that the estimated prevalence will be between 3.6% and 6.4%.

Comparisons between populations: Using a continuous measure if one population has a mean eGFR 5 mL/min lower than that in another population, the study will have more than 95% power to detect this difference; for comparisons between population subgroups (e.g. three subgroups of equal size), the study will have more than 80% power to detect a difference of 7 mL/min in mean eGFR. If one centre has a prevalence of impaired kidney function (eGFR <90 mL/min) of 5% and another centre has double the risk (i.e. prevalence of 10%), then a sample size of 1,000 will provide 99% power to detect this difference; for comparisons within centres, if the centre participants are divided into three equal groups, the study will have approximately 80% power to detect a doubling of risk when comparing any two of the three subgroups.

### Future plans

Prevalence studies are the basis on which the populations and study design for further investigations can be determined. Once variation in eGFR distribution and estimates of CKD and CKDu prevalence have been established, appropriately designed aetiological studies can be pursued. In collaboration with other investigators we will continue to develop protocols for follow-up studies (examining the roles of occupational and environmental factors) and intervention studies in affected populations.

## Discussion

### Weaknesses

This study involves cross-sectional surveys rather than repeated measures. Thus, it can identify population patterns, but cannot be used to diagnose CKDu in individuals, since the clinical diagnosis of CKD by impaired kidney function requires estimation of eGFR and proteinuria on two occasions at least 3 months apart (as a single measurement does not exclude an acute kidney injury; AKI). However, most participants are unlikely to be acutely unwell in a population sample, and therefore there is little evidence that AKI cases would affect the eGFR distribution as a whole in the study sample.

As mentioned above, the major challenges of performing international CKD comparisons are those of standardisation of serum creatinine measurements and those surrounding the differential bias using GFR estimating equations when comparing study participants of different ethnicities. With regard to GFR estimation this is likely to be a particular problem when characterising the upper end of the GFR distribution of the population rather than estimating the prevalence of advanced CKD. However, as international comparisons of advanced CKD alone would require prohibitively large sample sizes we have elected to compare the entire eGFR distribution between populations.

With recent international efforts the obstacle of lack of serum creatinine assay standardisation between laboratories has been largely overcome, however there are no validated methods to address the problem of differential bias in eGFR equations suitable for epidemiological studies. In an attempt to address what are likely to be the largest contributor to differential bias inherent in GFR estimating equations the DEGREE protocol includes both sample collection for future cystatin C measurements and data collection on body composition and self-reported meat consumption. Whole body bioimpedance is a simple but reasonable indicator of lean mass index and should allow a more robust comparison of eGFR between populations. Once improved estimating equations validated in the relevant populations and/or standardised cystatin C measurements become available, re-analysis of datasets will be performed.

Given the resource implications of undertaking formal albumin:creatinine ratios, we have opted for the simplest option, dipstick urinalysis, in the core protocol. Previous large-scale epidemiologic studies have demonstrated the predictive value of urine dipstick measurements using electronic readers (and where possible we advise these to be used) [29]. However if resources are available to store urine samples the possibility of measuring albumin:creatinine ratios could be explored.

### Governance

The DEGREE organisational structure comprises a Steering Committee (including the authors of this protocol) coordinated by an Executive (chaired by the first and last authors of this paper, and also including co-authors KJ, JG, AS and RC-R). A Reference Group will provide advice to the Steering Committee. Regional coordinators will be responsible for identifying lead investigators and facilitating local investigators. These coordinators will be encouraged to visit study sites. Expressions of interest for the reference group, and regional coordinators should be directed to the DEGREE Study Executive. In general, publications on international comparisons will be authored by the DEGREE Study Group, comprising members of the Steering Committee, coordinators, and a co-investigator from each participating centre.

The DEGREE executive is committed to maximizing the use of a common protocol to allow robust international comparisons. To this end, the project has been/will be promoted, and the rationale underlying the protocol presented, at a number of international meetings including the 2015 Mesoamerican Nephropathy Workshop, 2016 Institute for Global Development India meeting on CKDnt, the 2016 World Health Organisation Workshop on CKDu in Sri Lanka and the 2016 Epidemiology in Occupational Health Conference (EPICOH). In addition the DEGREE collaboration will continue to work with key stakeholders, including but not limited to: the Consortium on the Epidemic of Nephropathy in Central America and Mexico, the International Society of Nephrology and the Inter-American Development Bank. Funding has been identified for engagement and communications and a DEGREE website, hosting protocols and other resources, will soon be available via the London School of Hygiene and Tropical Medicine Centre for Non-Communicable diseases (http://globalncds.lshtm.ac.uk). Most importantly however, the DEGREE collaboration will support local investigators with training and networking as well as assisting them in obtaining the necessary resources to conduct these studies.

### Additional modules

A number of further optional additional modules will be made available through the DEGREE Steering Committee. Specifically we plan to develop tools that allow occupational and environmental exposures to be captured using standardised tools.

## Conclusions

We have here presented the core DEGREE Study protocol for measuring population patterns of eGFR. We will develop additional modules for follow-up studies (e.g. examining the roles of occupational and environmental factors) and intervention studies in affected populations. Even in the first stage of DEGREE, the use of a common prevalence protocol will yield important information on population patterns and time trends, thus enabling us to gain a better understanding of the scope and scale of CKD, and the possible causes. This will provide a basis for further efforts to address the epidemic of kidney failure that is affecting disadvantaged populations around the world.

## Additional files

Additional file 1: DEGREE Centre Data. (DOC 154 kb)
Additional file 2: DEGREE Additional Optional Renal Protocol. (DOC 94 kb)
Additional file 3: DEGREE Core Protocol Proforma. (DOC 203 kb)
