# Supplementary material for: International Collaboration for the Epidemiology of eGFR in Low and Middle Income Populations - Rationale and core protocol for the Disadvantaged Populations eGFR Epidemiology Study (DEGREE)
Source: BMC Nephrol. 2017 Jan 3;18:1. doi: 10.1186/s12882-016-0417-1 (PMC5210224; doi:10.1186/s12882-016-0417-1)
Supplement: Additional file 1: — DEGREE Centre Data. (DOC 154 kb) [file 12882_2016_417_MOESM1_ESM.doc]

<insert country/site name/ID>

| DEGREE Centre Data |
| --- |

|  | |  | | |  |
| --- | --- | --- | --- | --- | --- |
| Lead Investigator |  | | | CNTR1 | |
| Contact Details | email | |  | CNTR2 | |
|  | telephone | |  | CNTR3 | |
|  | address | |  | CNTR4 | |
| Institution Responsible |  | | | CNTR5 | |
| IRB Approval Committee |  | | | CNTR6 | |
| IRB Approval Date |  | | | CNTR7 | |

| Geographic Location  *or Attach Map* | GIS coordinates |  | CNTR8a,b |
| --- | --- | --- | --- |
| Source of Study Population | Existing Census | 1 | CNTR9 |
|  | New Census | 2 |  |
| If existing census what date was this performed |  | └─┴─┘ └─┴─┴─┴─┘  dd mm year | CNTR10 |
| Sampling unit | Individual | 1 | CNTR11 |
|  | Household | 2 |  |
| Sample | Random | 1 | CNTR12 |
|  | Whole population | 2 |  |
| Randomisation method (if random sampling) |  | | CNTR13 |
| Start of study | └─┴─┘ └─┴─┴─┴─┘  dd mm year | | CNTR14 |
| End of study | └─┴─┘ └─┴─┴─┴─┘  dd mm year | | CNTR15 |

| Population and response |  | | | |  | |
| --- | --- | --- | --- | --- | --- | --- |
| Total population size (from census) |  | | | | CNTR16 | |
| Planned sample size |  | | | | CNTR17 | |
| Overall response rate | % | | | CNTR18 | | |
| **Women** (n) | Census | Sample | Responders | | |  |
| Age 18-30 |  |  |  | | | CNTR19a-c |
| Age 31-45 |  |  |  | | | CNTR20a-c |
| Age 46-60 |  |  |  | | | CNTR21a-c |
| Age >60 |  |  |  | | | CNTR22a-c |
| **Men** (n) | Census | Sample | Responders | | |  |
| Age 18-30 |  |  |  | | | CNTR23a-c |
| Age 31-45 |  |  |  | | | CNTR24a-c |
| Age 46-60 |  |  |  | | | CNTR25a-c |
| Age >60 |  |  |  | | | CNTR26a-c |

| Primary Industries | Subsistence Agriculture | 1 | CNTR27 |
| --- | --- | --- | --- |
|  | Commercial Agriculture | 2 |  |
|  | Mining | 3 |  |
|  | Fishing | 4 |  |
|  | Manufacturing | 5 |  |
|  | Services | 6 |  |
| Predominant Crops |  | |  |
| Approximate Median Income |  | $ |  |
| Typical Water Sources | Piped | 1 | CNTR28 |
|  | Deep well | 2 |  |
|  | Shallow well | 3 |  |
|  | Surface water | 4 |  |
| Healthcare System |  |  |  |
| Availability of RRT | Publically funded dialysis | 1 | CNTR29 |
|  | Private dialysis only | 2 |  |
|  | Publically funded transplantation | 3 |  |
|  | Private transplantation only | 4 |  |
|  | No accessible renal replacement therapy | 5 |  |

| LABORATORY DETAILS |  | |  | | | |  |
| --- | --- | --- | --- | --- | --- | --- | --- |
| Sample biobanking and storage |  | | tick and storage temperature | | | |  |
|  | Urine | | ☐ └─┴─┘ | | | | CNTR30 |
|  | Whole blood | | ☐ └─┴─┘ | | | | CNTR31 |
|  | Serum | | ☐ └─┴─┘ | | | | CNTR32 |
| Contact details of laboratory for creatinine |  | | | | | | CNTR33 |
| Dates samples analysed | └─┴─┘ └─┴─┴─┴─┘ to └─┴─┘ └─┴─┴─┴─┘  dd mm year dd mm year | | | | | | CNTR34 |
| Instrument (manufacturer and model) |  | | | | | | CNTR35 |
| Creatinine standard (product and manufacturer) |  | | | | | | CNTR36 |
| Frequency of QC |  | | | | | | CNTR37 |
| Date of most recent machine QC prior to analysis | └─┴─┘ └─┴─┴─┴─┘  dd mm year | | | | | | CNTR38 |
| QC results | Creatinine Concentration of Standard | |  |  |  |  |  |
|  | CVa  Analytical coefficient of variation [SD/mean*100] | |  |  |  |  | CNTR39a-d |
|  | Bias  [Measured sCr – Standard concentration] * 100 | |  |  |  |  | CNTR40a-d |
| Are urinalysis strips measured using an electronic reader? | Yes | 1 | | | | | CNTR41 |
|  | No | 2 | | | | |  |
| DATA MANAGEMENT DETAILS |  |  | | | | |  |
| Data entry method | Manual | 1 | | | | | CNTR42 |
|  | Direct electronic entry | 2 | | | | |  |
| Double data entry? | Yes | 1 | | | | | CNTR43 |
|  | No | 2 | | | | |  |
| Have barcoded labels been used on questionnaires and samples? | Yes | 1 | | | | | CNTR44 |
|  | No | 2 | | | | |  |
