# Supplementary material for: International Collaboration for the Epidemiology of eGFR in Low and Middle Income Populations - Rationale and core protocol for the Disadvantaged Populations eGFR Epidemiology Study (DEGREE)
Source: BMC Nephrol. 2017 Jan 3;18:1. doi: 10.1186/s12882-016-0417-1 (PMC5210224; doi:10.1186/s12882-016-0417-1)
Supplement: Additional file 2: — DEGREE Additional Optional Renal Protocol. (DOC 94 kb) [file 12882_2016_417_MOESM2_ESM.doc]

| DEGREE Additional Optional Renal Protocol |
| --- |

| Question | Response | | Code |
| --- | --- | --- | --- |
| Has a doctor diagnosed you with kidney disease? | No | 1 *go to question KI3* | KI1 |
|  | Yes | 2 *go to question KI2* |  |
| Have you been told you have one of these kidney disease? | Glomerulonephritis | 1 | KI2 |
|  | Congenital abnormality of the kidneys | 2 |  |
|  | Polycystic kidney disease | 3 |  |
|  | Diabetic kidney disease | 4 |  |
|  | [locally defined] | 5 |  |
|  | [locally defined] | 6 |  |
|  | [locally defined] | 7 |  |
|  | Not told a cause | 8 |  |
| Have you been told you have ever been told you have one of these diseases? | Tuberculosis | 1 | KI3 |
|  | HIV | 2 |  |
|  | Hepatitis B | 3 |  |
|  | Hepatitis C | 4 |  |
|  | Schistosomiasis | 5 |  |
|  | Leptospirosis | 6 |  |
|  | [locally defined] | 7 |  |
|  | [locally defined] | 8 |  |
| Do you take herbal or traditional remedies? | No | 1 | KI4 |
|  | Yes | 2 |  |
| Do you take regular prescribed medications? | No | 1 *go to question KI 9* | KI5 |
|  | Yes | 2  *go to questions below* |  |
| Do you take medication for diabetes? | No | 1 | KI6 |
|  | Yes | 2 |  |
| Do you take medication against HIV or hepatitis? | No | 1 | KI7 |
|  | Yes | 2 |  |
| Do you take medication for tuberculosis? | No | 1 | KI8 |
|  | Yes | 2 |  |
| Have you used painkillers most days for more than several months ?  [*Use Showcard with locally avaliable medications*]? | Yes | 1 | KI9 |
|  | No | 2 |  |

| DEGREE study optional example additional testing |
| --- |

| Ultrasound |  |  |  |
| --- | --- | --- | --- |
| Device ID for ultrasound |  | └─┴─┴─┘ | OP1 |
| Ultrasound Investigator ID |  | └─┴─┴─┘ | OP2 |
| Kidney size right  *enter 00 for not seen/missing* | *XX.X cm* | └─┴─┴─┘cm | OP3 |
| Kidney size left  *enter 00 for not seen/missing* | *XX.X cm* | └─┴─┴─┘cm | OP4 |
| Is there hydronephrosis? | No | 1 | OP5 |
|  | Yes | 2 |  |
| Are there renal calculi? | No | 1 | OP6 |
|  | Yes | 2 |  |
| Are there signs of renal scarring | No | 1 | OP7 |
|  | Yes | 2 |  |
| Other abnormalities [free text] |  |  | OP8 |

| Additional optional laboratory tests |  |  |  |
| --- | --- | --- | --- |
| For blood glucose:  Is this a fasting sample? | No | 1 | OP9 |
|  | Yes | 2 |  |
| Blood Glucose  *[CHOOSE ACCORDINGLY:* mmol/l *OR mg/dl]* | mmol/l | └─┴─┴─┘ | OP10 |
|  | mg/dl | └─┴─┴─┘ | OP11 |
| Uric acid (Urate)  *[CHOOSE ACCORDINGLY: μmol/l OR mg/dl]* | mmol/l | └─┴─┴─┘ | OP12 |
|  | mg/dl | └─┴─┴─┘ | OP13 |
| Urine albumin creatinine ratio  *[CHOOSE ACCORDINGLY: mg/mmol OR μg/mg]* | mg/mmol | └─┴─┴─┘ | OP14 |
|  | μg/mg | └─┴─┴─┘ | OP15 |
| Additional testing as per local investigators | | | |
