# Supplementary material for: International Collaboration for the Epidemiology of eGFR in Low and Middle Income Populations - Rationale and core protocol for the Disadvantaged Populations eGFR Epidemiology Study (DEGREE)
Source: BMC Nephrol. 2017 Jan 3;18:1. doi: 10.1186/s12882-016-0417-1 (PMC5210224; doi:10.1186/s12882-016-0417-1)
Supplement: Additional file 3: — DEGREE Core Protocol Proforma. (DOC 203 kb) [file 12882_2016_417_MOESM3_ESM.doc]

| DEGREE Core Protocol Porforma |
| --- |

|  | | Response | | Code | |
| --- | --- | --- | --- | --- | --- |
| Study site ID | └─┴─┴─┘ | | I1 | |  |
| Interviewer ID | └─┴─┴─┘ | | I2 | |  |
| Date of completion of the instrument | └─┴─┘ └─┴─┘ └─┴─┴─┴─┘ dd mm year | | I3 | |  |

| Consent, Interview Language and Name | Response | | Code |
| --- | --- | --- | --- |
| Consent has been read and obtained | Yes | 1 | I5 |
|  | No | 2 If NO, END |  |
| Interview Language *[Insert Language]* | English | 1 | I6 |
|  | *[Add others]* | 2 |  |
|  | *[Add others]* | 3 |  |
|  | *[Add others]* | 4 |  |
| Time of interview  (24 hour clock) | └─┴─┘: └─┴─┘  hrs mins | | I7 |
| Family Surname |  | | I8 |
| First Name |  | | I9 |
| Additional Information that may be helpful | | | |
| Contact phone number where possible |  | | I10 |

| DEGREE study core participant questionnaire (adapted from WHO STEPwise) |
| --- |

| Question | Response | | | | Code |
| --- | --- | --- | --- | --- | --- |
| Sex (*Record Male / Female as observed)* | Male | | 1 | | C1 |
|  | Female | | 2 | |  |
| What is your date of birth?  *Don't Know 77 77 7777* | dd mm year | | | | C2 |
| How old are you? | Years | |  | | C3 |
| In total, how many years have you spent at school and in full-time study (excluding pre-school)? | Years |  | | | C4 |
| What is your *[insert relevant ethnic group / racial group / cultural subgroup / others]* background? | [*Locally defined]* | 1 | | | C6 |
|  | [*Locally defined]* | 2 | | |  |
|  | [*Locally defined]* | 3 | | |  |
|  | Refused | 88 | | |  |
| Which of the following best describes your main work status over the past 12 months? | Government employee | 1 | | | C8 |
|  | Non-government employee | 2 | | |  |
|  | Self-employed | 3 | | |  |
|  | Non-paid | 4 | | |  |
|  | Student | 5 | | |  |
|  | Homemaker | 6 | | |  |
|  | Retired | 7 | | |  |
|  | Unemployed (able to work) | 8 | | |  |
|  | Unemployed (unable to work) | 9 | | |  |
|  | Unpaid domestic | 10 | | |  |
|  | Refused | 88 | | |  |
| If you are working what is your main occupation *[FREE TEXT]:* |  |  | | | OCCTXT |
| Do you have experience of migrant work?  [Defined as staying far from home for seasonal work] | Yes | | | 1 | MIGR |
|  | No | | | 2 |  |
| Can you give an estimate of the monthly household income if I read some options to you? Is it  *[INSERT QUINTILE VALUES IN LOCAL CURRENCY]*  *(READ OPTIONS)* | ≤ Quintile (Q) 1 | 1 | | | C11 |
|  | More than Q 1, ≤ Q 2 | 2 | | |  |
|  | More than Q 2, ≤ Q 3 | 3 | | |  |
|  | More than Q 3, ≤ Q 4 | 4 | | |  |
|  | More than Q 4 | 5 | | |  |
|  | Don't Know | 77 | | |  |
|  | Refused | 88 | | |  |
| During the past 12 months, how frequently have you had at least one standard alcoholic drink?  *(READ RESPONSES, USE SHOWCARD)* | Daily | 1 | | | A4 |
|  | 5-6 days per week | 2 | | |  |
|  | 3-4 days per week | 3 | | |  |
|  | 1-2 days per week | 4 | | |  |
|  | 1-3 days per month | 5 | | |  |
|  | Less than once a month | 6 | | |  |
|  | Not at all | 7 | | |  |
|  | Refused | 88 | | |  |
| Do you currently smoke any tobacco products, such as cigarettes, cigars or pipes? *(USE SHOWCARD)* | Yes | 1 | | | *T1* |
|  | No | 2 | | |  |
| In a typical week, on how many days do you eat MEAT *(USE SHOWCARD)* | Number of days Don't Know 77 | └─┴─┘ | | | *D1* |
| Does your work involve vigorous-intensity activity that causes large increases in breathing or heart rate like *[carrying or lifting* *heavy loads, digging or construction work]*  for at least 10 minutes continuously? *(OR USE SHOWCARD)* | Yes | 1 | | | P1 |
|  | No | 2 | | |  |
| Have you ever been told by a doctor or other health worker that you have raised blood pressure or hypertension? | Yes | 1 | | | H2a |
|  | No | 2 | | |  |
| Have you ever been told by a doctor or other health worker that you have raised blood sugar or diabetes? | Yes | 1 | | | H7a |
|  | No | 2 | | |  |

| DEGREE study core physical measurements |
| --- |

| Question | Response | | | Code |
| --- | --- | --- | --- | --- |
| Ambient Temperature  (at time of examination measured in shade) |  | | └─┴─┴─┘**°**C | Temp |
| Blood Pressure | | | | |
| Question | Response | | | Code |
| Interviewer ID |  | └─┴─┴─┘ | | M1 |
| Device ID for blood pressure |  | └─┴─┴─┘ | | M2 |
| Cuff size used | Small | 1 | | M3 |
|  | Medium | 2 | |  |
|  | Large | 3 | |  |
| Reading 1 | Systolic ( mmHg) | └─┴─┴─┘ | | M4a |
|  | Diastolic (mmHg) | └─┴─┴─┘ | | M4b |
| Reading 2 | Systolic ( mmHg) | └─┴─┴─┘ | | M5a |
|  | Diastolic (mmHg) | └─┴─┴─┘ | | M5b |
| Reading 3 | Systolic ( mmHg) | └─┴─┴─┘ | | M6a |
|  | Diastolic (mmHg) | └─┴─┴─┘ | | M6b |
| During the past two weeks, have you been treated for raised blood pressure with drugs (medication) prescribed by a doctor or other health worker? | Yes | 1 | | M7 |
|  | No | 2 | |  |
| Height, Weight and BIA | | | | |
| For women: Are you pregnant? | Yes | 1 | | M8 |
|  | No | 2 | |  |
| Have you eaten yet today? | Yes | 1 | | M9 |
|  | No | 2 | |  |
| Interviewer ID |  | └─┴─┴─┘ | | M10 |
| Height | in Centimetres (cm) | └─┴─┴─┘ | | M11 |
| Weight  *If too large for scale 666.6* | in Kilograms (kg) | └─┴─┴─┘ | | M12 |
| Device ID for BIA |  | └─┴─┴─┘ | | M13 |
| BIA Device make and model (50Hz supine) |  |  | | M14 |
| Bioimpedence value |  | └─┴─┴─┘ Ω | | BIA1 |
| Bioimpedance outputs in kg or % *(tick)* |  | ☐kg ☐% | | BIA2 |
| Bioimpedance outputs | Fat Mass  OR | └─┴─┴─┘ | | BIA3a |
| *different machines may provide different outputs* | Fat Free Mass  (also termed lean mass) | └─┴─┴─┘ | | BIA3b |

** if bioimpedence data is not collected please contact the DEGREE Study Committee prior to commencing the study

| DEGREE study core lab measurements |
| --- |

| Question | Response | | Code |
| --- | --- | --- | --- |
| Blood sampling Investigator ID |  | └─┴─┴─┘ | B2 |
| Time of day blood specimen taken (24 hour clock) | Hours : minutes | └─┴─┘: └─┴─┘  hrs mins | B4 |
| Creatinine measurement Technician ID |  | └─┴─┴─┘ | CR1 |
| Creatinine measurement Device ID |  | └─┴─┴─┘ | CR2 |
| Serum Creatinine | *to first decimal place if in mg/dL* | └─┴─┴─┘ | CR3 |
| Serum Creatinine Units | mg/dL | 1 | CR4 |
|  | μMol/L | 2 |  |
| Urine sampling Investigator ID |  | └─┴─┴─┘ | UR1 |
| Urinalysis Device ID |  | └─┴─┴─┘ | UR2 |
| Urine Glucose | Negative | 1 | UR3 |
|  | 100mg/dL | 2 |  |
|  | 250mg/dL | 3 |  |
|  | 500mg/dL | 4 |  |
|  | 1000mg/dL | 5 |  |
|  | >2000mg/dL | 6 |  |
| Urine Specific Gravity | 1.000 | 1 | UR4 |
|  | 1.005 | 2 |  |
|  | 1.010 | 3 |  |
|  | 1.015 | 4 |  |
|  | 1.020 | 5 |  |
|  | 1.025 | 6 |  |
|  | 1.030 | 7 |  |
| Urinalysis Blood | Negative | 1 | UR5 |
|  | Non-haemolysed trace | 2 |  |
|  | Non-haemolysed moderate | 3 |  |
|  | Haemolysed trace | 4 |  |
|  | Small (+) | 5 |  |
|  | Moderate (++) | 6 |  |
|  | Large (+++) | 7 |  |
| Urine pH | 5.0 | 1 | UR6 |
|  | 6.0 | 2 |  |
|  | 6.5 | 3 |  |
|  | 7.0 | 4 |  |
|  | 7.5 | 5 |  |
|  | 8.0 | 6 |  |
|  | 8.5 | 7 |  |
| Urinalysis Protein | Negative | 1 | UR7 |
|  | Trace | 2 |  |
|  | 30mg/dL (+) | 3 |  |
|  | 100mg/dL (++) | 4 |  |
|  | 300mg/dL (+++) | 5 |  |
|  | >2000mg/dL | 6 |  |
| Urinalysis Nitrite | Negative | 1 | UR8 |
|  | Positive | 2 |  |
| Urinalysis Leucocytes | Negative | 1 | UR9 |
|  | Trace | 2 |  |
|  | Small (+) | 3 |  |
|  | Moderate (++) | 4 |  |
|  | Large (+++) | 5 |  |
